# In-depth investigation of microRNA-mediated cross-kingdom regulation between Asian honey bee and microsporidian

**DOI:** 10.3389/fmicb.2022.1003294

**Published:** 2022-09-29

**Authors:** Xiaoxue Fan, Wende Zhang, Kaiyao Zhang, Jiaxin Zhang, Qi Long, Ying Wu, Kuihao Zhang, Leran Zhu, Dafu Chen, Rui Guo

**Affiliations:** ^1^College of Animal Sciences (College of Bee Science), Fujian Agriculture and Forestry University, Fuzhou, Fujian, China; ^2^Apitherapy Research Institute, Fujian Agriculture and Forestry University, Fuzhou, Fujian, China

**Keywords:** honey bee, *Apis cerana cerana*, *Nosema ceranae*, microsporidian, regulation network, infection mechanism

## Abstract

Asian honey bee *Apis cerana* is the original host for *Nosema ceranae*, a unicellular fungal parasite that causes bee nosemosis throughout the world. Currently, interaction between *A. cerana* and *N. ceranae* is largely unknown. Our group previously prepared *A. c. cerana* workers’ midguts at 7 days post inoculation (dpi) and 10 dpi with *N. ceranae* spores as well as corresponding un-inoculated workers’ midguts, followed by cDNA library construction and a combination of RNAs-seq and small RNA-seq. Meanwhile, we previously prepared clean spores of *N. ceranae*, which were then subjected to cDNA library construction and deep sequencing. Here, based on the gained high-quality transcriptome datasets, *N. ceranae* differentially expressed mRNAs (DEmiRNAs) targeted by host DEmiRNAs, and *A. c. cerana* DEmRNAs targeted by microsporidian DEmiRNAs were deeply investigated, with a focus on targets involved in *N. ceranae* glycolysis/glyconeogenesis as well as virulence factors, and *A. c. cerana* energy metabolism and immune response. In *A. c. cerana* worker’s midguts at 7 (10) dpi (days post inoculation), eight (seven) up-regulated and six (two) down-regulated miRNAs were observed to target 97 (44) down-regulated and 60 (15) up-regulated *N. ceranae* mRNAs, respectively. Additionally, two up-regulated miRNAs (miR-60-y and miR-676-y) in host midgut at 7 dpi could target genes engaged in *N. ceranae* spore wall protein and glycolysis/gluconeogenesis, indicating potential host miRNA-mediated regulation of microsporidian virulence factor and energy metabolism. Meanwhile, in *N. ceranae* at 7 (10) dpi, 121 (110) up-regulated and 112 (104) down-regulated miRNAs were found to, respectively, target 343 (247) down-regulated and 138 (110) down-regulated mRNAs in *A. c. cerana* workers’ midguts. These targets in host were relevant to several crucial cellular and humoral immune pathways, such as phagasome, endocytosis, lysosomes, regulation of autophagy, and Jak–STAT signaling pathway, indicative of the involvement of *N. ceranae* DEmiRNAs in regulating these cellular and humoral immune pathways. In addition, *N. ceranae* miR-21-x was up-regulated at 7 dpi and had a target relative to oxidative phosphorylation, suggesting that miR-21-x may be used as a weapon to modulate this pivotal energy metabolism pathway. Furthermore, potential targeting relationships between two pairs of host DEmiRNAs-microsporidian DEmRNAs and two pairs of microsporidian DEmiRNAs-host DEmRNAs were validated using RT-qPCR. Our findings not only lay a foundation for exploring the molecular mechanism underlying cross-kingdom regulation between *A. c. cerana* workers and *N. ceranae*, but also offer valuable insights into Asian honey bee-microsporidian interaction.

## Introduction

*Apis cerana cerana*, a subspecies of the Asian honey bee *Apis cerana*, is widely reared in China and many other Asian countries (Wu et al., 2020). Compared with the western honey bee *Apis mellifera*, *A. cerana* has several advantages, such as extreme weather condition adaptations, sporadic nectar source collection, hygienic behavior, and colony-level defense ability, and it is therefore of special economic and ecological value (Arias and Sheppard, 2005; Gallai et al., 2009; Hepburn and Radloff, 2011). The reference genome of *A. cerana* was published in 2015 (Pelin et al., 2015), which provides a key basis for further investigation of its biology and the underlying molecular mechanisms (Park et al., 2015).

*Nosema ceranae* is an intracellular fungal parasite that infects the midgut epithelial cells of adult bees (Traver and Fell, 2011, 2012) and bee larvae (Eiri et al., 2015). *N. ceranae* infection could damage the host midgut cell structure, cause energy stress, immunosuppression, and cell apoptosis inhibition, and influence bee health and colony productivity in combination with other biotic or abiotic stresses (Paris et al., 2018). The transmission of *N. ceranae* among individuals occurs mainly through the feces-oral or oral-oral route. A single coiled polar filament is highly compacted around the interior of the *N. ceranae* spore, and upon stimulation by the environment within the bee host midgut, the fungal spore germinates and then the polar tube ejects, which is then pierces into the host cell, followed by injection of infective sporaplasm into the host cell and initiation of the proliferation stage (Mayack et al., 2015; Martín-Hernández et al., 2018). Similar to other microsporidia, after long-term evolution and coadaptation, *N. ceranae* has evolved an extremely reduced genome with a very small size and lost the majority of pathways relevant to material and energy metabolism, such as the TCA cycle and oxidative phosphorylation (Cornman et al., 2009). Hence, *N. ceranae* proliferation is highly dependent on the host cell-derived material and energy (Paris et al., 2018). Increasing evidence indicates that *N. ceranae* could enhance the synthesis of amino acids, lipids, and nucleotides through releasing synthetic hexokinase into host cells and facilitate their proliferation by inhibiting host cell apoptosis (Cuomo et al., 2012).

An array of transcriptomic studies has been performed to analyze the response of *A. mellifera* workers to *N. ceranae* invasion. For example, based on transcriptomic investigation of the immune response of *A. m. ligustica* workers to *N. ceranae* infection, Fu et al. (2019) revealed that genes encoding antimicrobial peptides such as apideacin, defensin-1, and hymenoptaecin were differentially expressed during the fungal infection process. However, few omics studies have focused on *N. ceranae* in the infection process. To clarify the mechanisms of *N. ceranae* parasitism. Huang et al. (2016b) conducted deep sequencing and time-series analysis of *N. ceranae*-infected *A. mellifera* workers’ midgut tissues, the results showed that 1,122 microsporidian genes were clustered into four expression patterns and significantly differentially expressed during the reproduction cycle. By dissecting the transcriptomic dynamics of *N. ceranae* infecting *A. m. ligustica* workers, our group unraveled that genes encoding virulence factors, such as spore wall protein and ricin B lectin, were likely to play crucial roles in microsporidian proliferation (Geng et al., 2020b).

MicroRNAs (miRNAs) are single-stranded small noncoding RNAs (ncRNAs) with a length distribution of 19–25 nt, and they play a regulatory role in gene expression at the posttranscriptional level, leading to mRNA cleavage or translational suppression (Garofalo and Croce, 2011). MiRNAs have been suggested to participate in a substantial quantity of biological processes, such as cell differentiation and immune response (Bartel, 2004; Pillai et al., 2005). Recent documentation has demonstrated that miRNAs can not only regulate endogenous gene expression but also modulate the expression of exogenous genes. Zhang et al. (2012) discovered that plant-derived MIR168a and MIR156a were stably expressed in humans and mice and negatively regulated the expression of the target gene encoding LDLRAP1, and their work was the first on miRNA-mediated cross-kingdom regulation between plants and animals. Thereafter, an increasing number of studies confirmed cross-kingdom regulation among animals, plants, and microorganisms (Liu et al., 2012; Zhang et al., 2016; Cui et al., 2019). However, studies on cross-regulation between bees and pathogens are still very limited. After silencing the *Dicer* gene in *N. ceranae* with specific siRNA, Evans and Huang (2018) performed next-generation sequencing and analysis of *A. mellifera* workers and *N. ceranae* across a full fungal proliferation cycle, and the results suggested that *N. ceranae* miRNAs may regulate the expression of genes in both parasites and hosts. Recently, using transcriptome sequencing and bioinformatics, our team conducted a comprehensive investigation of miRNA-mediated regulation between *A. m. ligustica* workers and *N. ceranae* during the infection process (Du et al., 2021; Fan et al., 2021).

Currently, miRNA-mediated cross-kingdom regulation between eastern honey bees and microsporidians is completely unknown. Here, for the first time, based on previously obtained high-quality transcriptome data, differentially expressed mRNAs (DEmRNAs) in microsporidians targeted by *A. c. cerana* DEmiRNAs and host DEmRNAs targeted by *N. ceranae* DEmiRNAs were predicted and analyzed, followed by an in-depth investigation of DEmiRNA-mediated cross-kingdom regulation between hosts and microsporidians. Our results will not only lay a key foundation for clarifying the mechanism underlying miRNA-mediated cross-kingdom regulation between *A. c. cerana* and *N. ceranae*, but also offer new insight into interactions between Asian honey bee and microsporidian.

## Materials and methods

### Source of sRNA-seq and RNA-seq data from *Apis cerana cerana* workers’ midguts

In our previous work, midgut tissues of *N. ceranae*-inoculated *A. c. cerana* workers at 7 days post-inoculation (dpi) and 10 dpi (AcTmi7 group: AcTmi7-1, AcTmi7-2, AcTmi7-3; AcTmi10 group: AcTmi10-1, AcTmi10-2, AcTmi10-3) and corresponding un-inoculated workers’ midgut tissues (AcCKmi7 group: AcCKmi7-1, AcCKmi7-2, AcCKmi7-3; AcCKmi10 group: AcCKmi10-1, AcCKmi10-2, AcCKmi10-3) were prepared. Briefly, newly emerged *Nosema*-free workers were carefully removed from frames in three *A. c. cerana* colonies, which were raised in College of Animal Sciences (College of Bee Science), and then transfered to plastic cages in groups of 30, and reared in an incubator at 34 ± 2°C for 24 h; the workers in treatment groups (*n* = 3) were starved for 2 h and then each was fed 5 μl of a 50% sucrose (w/v in sterile water) solution containing 1 × 10^6^ *N. ceranae* spores, whereas workers in control groups (*n* = 3) were each fed 5 μl of a 50% sucrose solution without spores; at 24 h after inoculation, the workers were fed *ad libitum* with a sucrose solution and the feeders were replaced daily; nine workers from each cage in the *N. ceranae*-treated and control groups were sacrificed at 7 d post-inoculation (dpi) and 10 dpi, and the midgut tissues were then dissected and immediately frozen in liquid nitrogen. cDNA library construction was conducted followed by deep sequencing utilizing sRNA-seq technology (Du et al., 2019). Raw data were deposited in the Sequence Read Archive (SRA) database^1^ and connected to BioProject: PRJNA487111.

In another previous study, midgut tissues of *N. ceranae*-inoculated *A. c. cerana* workers at 7 dpi (AcTm7 group: AcTm7-1, AcTm7-2, AcTm7-3) and 10 dpi (AcTm10 group: AcTm10-1, AcTm10-2, AcTm10-3) and corresponding un-inoculated workers’ midgut tissues (AcCKm7 group: AcCKm7-1, AcCKm7-2, AcCKm7-3; AcCKm10: AcCKm10-1, AcCKm10-2, AcCKm10-3) were prepared according to the protocol mentioned above, followed by strand-specific cDNA library construction and deep sequencing using RNA-seq technology (Xing et al., 2021).

### Sources of sRNA-seq and RNA-seq data from *Nosema ceranae* spores

*Nosema ceranae* spores (NcCKmi group: NcCKmi-1, NcCKmi-2, and NcCKmi-3) were previously prepared with the Percoll discontinuous density gradient centrifugation method by our group, followed by cDNA library construction and sRNA-seq (Geng et al., 2020a). Raw data from sRNA-seq were uploaded to the NCBI SRA database under BioProject number PRJNA395264. Meanwhile, the prepared *N. ceranae* spores (NcCKm group: NcCKm-1, NcCKm-2, and NcCKm-3) were subjected to cDNA library construction and RNA-seq (Guo et al., 2018) Original data were uploaded to the NCBI SRA database and linked to BioProject number PRJNA395264.

### Source of sRNA-seq and RNA-seq data from *Nosema ceranae* infecting *Apis cerana cerana* workers

Based on our established protocol, clean tags from *N. ceranae* infecting *A. c. cerana* workers at 7 dpi and 10 dpi (NcTmi7 group: NcTmi7-1, NcTmi7-2, NcTmi7-3; NcTmi10 group: NcTmi10-1, NcTmi10-2, NcTmi10-3) were filtered out from the sRNA-seq data derived from *A. c. cerana* workers’ midgut tissues at 7 dpi and 10 dpi (Du et al., 2019). In brief, (1) the clean tags from the sRNA-seq of the midgut tissues of *A. c. cerana* workers at 7 dpi and 10 dpi were first mapped to the GenBank and Rfam databases to remove ribosomal RNA (rRNA), small cytoplasmic RNA (scRNA), small nucleolar RNA (snoRNA), small intranuclear RNA (snRNA), and transport RNA (tRNA) data; (2) the unmapped clean reads were then mapped to the *A. cerana* reference genome (Assembly ACSNU-2.0) by using Bowtie software (Langmead et al., 2009) to remove host-derived data; and (3) the unmapped clean tags were further mapped to the *N. ceranae* reference genome (Assembly ASM98816v1), while the mapped data were derived from *N. ceranae* during the infection. Original data were uploaded to the NCBI SRA database and linked to BioProject number PRJNA406998.

Following our previously described method, clean reads from *N. ceranae* infecting *A. c. cerana* workers at 7 dpi and 10 dpi (NcTm7 group: NcTm7-1, NcTm7-2, NcTm7-3; NcTm10 group: NcTm10-1, NcTm10-2, NcTm10-3) were filtered out from the RNA-seq data derived from *A. c. cerana* workers’ midgut tissues at 7 dpi and 10 dpi (Xiong et al., 2020). Original data were uploaded to the NCBI SRA database and linked to BioProject number PRJNA562784.

### Prediction and analysis of *Nosema ceranae* DEmRNAs targeted by *Apis cerana cerana* DEmiRNAs

The expression level of each *A. c. cerana* miRNA was normalized to the total number of sequence tags per million (TPM) based on the following formula: normalized expression = mapped read count/total reads × 10^6^. edgeR software (Robinson et al., 2010) was used to screen out the DEmiRNAs in AcCKmi7 vs. AcTmi7 and AcCKmi10 vs. AcTmi10 comparison groups following the criteria of *p* value (FDR corrected) < 0.05 and |log_2_(Fold change)| > 1.

The expression level in each *N. ceranae* mRNA was normalized to the fragments per kilobase of transcript per million fragments mapped (FPKM) based on the following formula: total exon fragments/[mapped reads (millions) × exon length (KB)]. DEmRNAs in the NcCKm vs. NcTm7 and NcCKm vs. NcTm10 comparison groups were screened out based on the criteria of *p* value (FDR corrected) < 0.05 and |log_2_(Fold change)| > 1.

*N. ceranae* DEmRNAs targeted by *A. c. cerana* DEmiRNAs were predicted using TargetFinder software (Allen et al., 2005) with the default parameters. Gene Ontology (GO) classification and Kyoto Encyclopedia of Genes and Genomes (KEGG) pathway analysis of the aforementioned *A. c. cerana* DEmiRNAs were performed using related tools in the OmicShare platform.^2^ Regulatory networks between *A. c. cerana* DEmiRNAs and *N. ceranae* DEmRNAs were constructed based on targeting relationships followed by visualization using Cytoscape v.3.2.1 software (Smoot et al., 2011) with default parameters.

### Prediction and investigation of *Apis cerana cerana* DEmRNAs targeted by *Nosema ceranae* DEmiRNAs

DEmiRNAs in the NcCKmi vs. NcTmi7 and NcCKmi vs. NcTmi10 comparison groups and DEmRNAs in the NcCKm vs. NcTm7 and NcCKm vs. NcTm10 comparison groups were identified following the abovementioned methods.

*A. c. cerana* DEmRNAs targeted by *N. ceranae* DEmiRNAs were predicted with TargetFinder software. GO categorization and KEGG pathway analysis of *A. c. cerana* DEmRNAs were conducted using the OmicShare platform. Regulatory networks between *N. ceranae* DEmiRNAs and *A. c. cerana* DEmRNAs were constructed based on the targeting relationship and then visualized with Cytoscape v.3.2.1 software.

### RT–qPCR validation of DEmRNAs

To verify the reliability of the transcriptome datasets used in this study, according to the targeted binding relationship, two host DEmRNAs (XM_017055873.1, XM_017064721.1) in AcCKm7 vs. AcTm7, two microsporidian DEmiRNAs (miR-8,462-x, miR-676-y) in NcCKmi vs. NcTmi7, two microsporidian DEmRNAs (XM_002995668.1, XM_002995068.1) in NcCKm vs. NcTm7 and two host DEmiRNAs in AcCKm7 vs. AcTm7 were randomly selected for RT–qPCR. The first cDNA strand was synthesized with the SuperScript first-strand synthesis system (Yeasen) according to the manufacturer’s protocol. Primers for qPCR were designed utilizing DNAMAN software and synthesized by Sangon Biotech Co., Ltd. (Shanghai). The housekeeping gene actin was used as an internal control. The RNA samples used as templates for RNA-seq were the same as those used for RT–qPCR, which was conducted on a QuanStudio Real-Time PCR System (Thermo Fisher, Waltham, MA, United States). The 20 μl PCR mixture contained 10 μl SYBR Green dye (Yeasen), 1 μl (10 μmol/l) specific forward primer, 1 μl (10 μmol/l) reverse primer, 1 μl (10 ng/μL) diluted cDNA, and 7 μl RNase free water. The cycling parameters were as follows: 95°C for 1 min, followed by 40 cycles at 95°C for 15 s, 55°C for 30 s, and 72°C for 45 s. The relative gene expression was calculated using the 2^−ΔΔCT^ method. The experiment was carried out times using three independent biological samples.

## Results

### Summary of omics data from host and microsporidian

After strict quality control, 122,104,443 clean tags were obtained from sRNA-seq of *N. ceranae*-inoculated and un-inoculated midgut samples, and the Pearson correlation coefficient between every biological replicate in each group was above 96.19% (Du et al., 2019); 1,562,162,742 clean reads with an average Q30 of 94.76% were gained from RNA-seq, and the Pearson correlation coefficient was above 0.87 (Xing et al., 2021).

### Analysis of *Nosema ceranae* DEmRNAs targeted by DEmiRNAs in *Apis cerana cerana* workers’ midguts and corresponding regulatory networks

**Figure 1:**
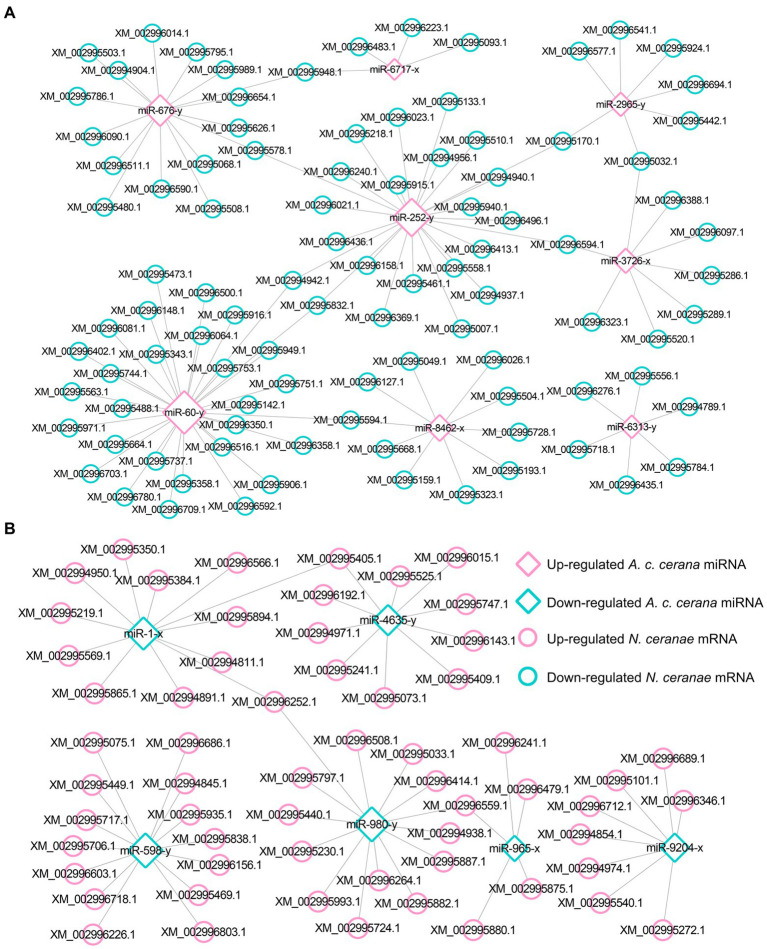
Regulatory network between *A. c. cerana* DEmiRNAs and target DEmRNAs in *N. ceranae* at 7 dpi. **(A)** Regulatory network between up-regulated miRNAs in host and down-regulated mRNAs in microsporidian. **(B)** Regulatory network between down-regulated miRNAs in host and up-regulated mRNAs in microsporidian.

Target prediction suggested that 97 down-regulated mRNAs in NcCKm vs. NcTm7 were potentially targeted by eight up-regulated miRNAs in AcCKmi7 vs. AcTmi7 (Figure 1A; see also Supplementary Table S1), whereas 60 up-regulated *N. ceranae* mRNAs were potential targets of six down-regulated *A. c. cerana* miRNAs (Figure 1B, see also Supplementary Table S1). The aforementioned 97 down-regulated mRNAs were related to eight biological process-associated terms, including metabolic process and cellular process; six cellular component-associated terms, including cell and cell part; and two molecular function-associated terms, including binding and catalytic activity (Supplementary Table S3). These down-regulated mRNAs were also annotated to 35 pathways, including metabolic pathways, biosynthesis of antibiotics, and biosynthesis of secondary metabolites (Supplementary Table S4). Additionally, the 60 up-regulated mRNAs were associated with 16 functional terms, such as metabolic process, cell part, and catalytic activity (Supplementary Table S3), and 33 pathways, such as metabolic pathway, biosynthesis of antibiotics, and biosynthesis of secondary metabolites (Supplementary Table S4).

**Figure 2:**
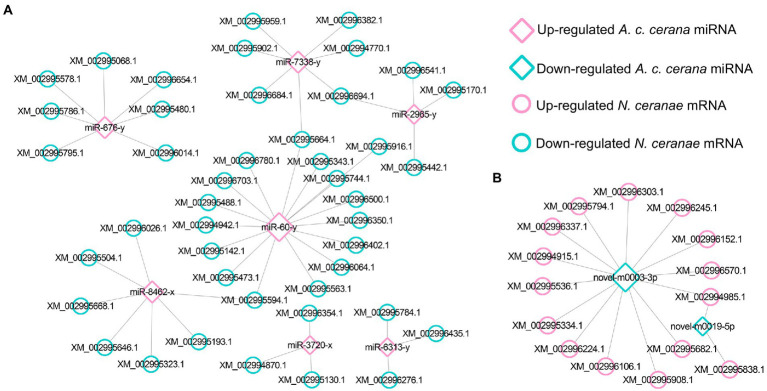
Regulatory network between *A. c. cerana* DEmiRNAs and target DEmRNAs in *N. ceranae* at 10 dpi. **(A)** Regulatory network between up-regulated miRNAs in host and down-regulated mRNAs in microsporidian. **(B)** Regulatory network between down-regulated miRNAs in host and up-regulated mRNAs in microsporidian.

In the NcCKm vs. NcTm10 comparison group, 44 down- and 15 up-regulated mRNAs were potentially targeted by seven up- and two down-regulated miRNAs in the AcCKmi10 vs. AcTmi10 comparison group, respectively (Figure 2; see also Supplementary Table S1). The abovementioned 15 up-regulated *N. ceranae* mRNAs were related to eight functional terms, including catalytic activity, binding, and metabolic processes (Supplementary Table S3), and 11 pathways, such as metabolic pathways, biosynthesis of secondary metabolites and carbon metabolism (Supplementary Table S4). Additionally, the 44 down-regulated *N. ceranae* mRNAs were engaged in eight functional terms, including metabolic process, cellular process, and single-organism process (Supplementary Table S3), and 24 pathways, including metabolic pathways, ribosome biogenesis in eukaryotes, and endocytosis (Supplementary Table S4).

**Figure 3:**
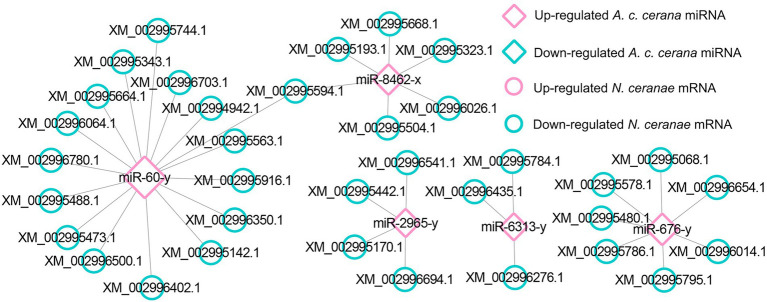
Regulatory network between up-regulated miRNAs shared by AcCKmi7 vs. AcTmi7 and AcCKmi10 vs. AcTmi10 and down-regulated mRNAs shared by NcCKm vs. NcTm7 and NcCKm vs. NcTm10.

Further investigation indicated that five up-regulated miRNAs shared by AcCKmi7 vs. AcTmi7 and AcCKmi10 vs. AcTmi10 comparison groups potentially targeted 35 down-regulated mRNAs shared by NcCKm vs. NcTm7 and NcCKm vs. NcTm10 comparison groups (Figure 3).

**Figure 4:**
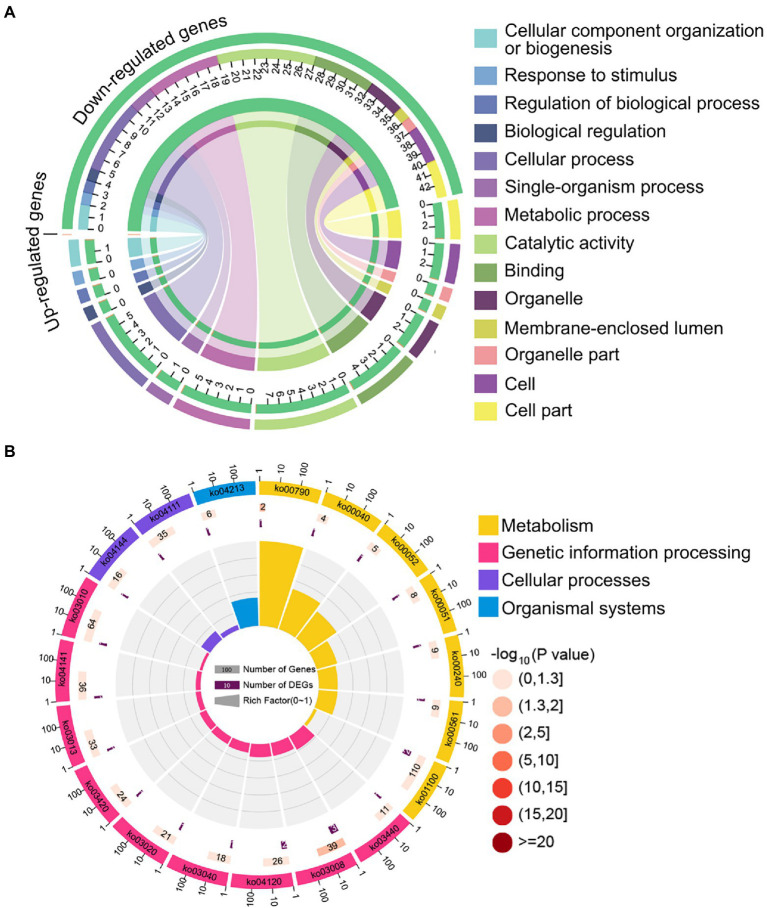
Database annotation of shared down-regulated *N. ceranae* mRNAs targeted by shared up-regulated *A. c. cerana* miRNAs. **(A)** GO database annotation of down-regulated mRNAs shared by NcCKm vs. NcTm7 and NcCKm vs. NcTm10. **(B)** KEGG database annotation of down-regulated mRNAs shared by NcCKm vs. NcTm7 and NcCKm vs. NcTm10.

The aforementioned common down-regulated *N. ceranae* mRNAs were annotated to 14 GO categories, including seven biological process-associated categories, including cellular processes and metabolic processes; five cellular component-associated categories, such as cell and cell part; and two molecular function-associated categories, such as catalytic activity and binding (Figure 4A). In addition, these common down-regulated mRNAs were annotated to 20 pathways, among which the most abundant was the metabolic pathway followed by ribosome biogenesis in eukaryotes and pyrimidine metabolism (Figure 4B).

### Investigation of *Apis cerana cerana* DEmiRNAs and their target DEmRNAs associated with *Nosema ceranae* virulence factors

In the AcCKmi7 vs. AcTmi7 comparison group, the up-regulated miR-60-y (log_2_FC = 10.87, *p* < 0.01) potentially targeted a spore wall protein-encoding gene (XM_002996592.1, log_2_FC = −2.24, *p* < 0.01) in the NcCKm vs. NcTm7 comparison group; and the up-regulated miR-676-y (log_2_FC = 12.97, *p* < 0.01) potentially targeted a gene encoding pyruvate dehydrogenase e1 component subunit alpha (XM_002996090.1, log_2_FC = −6.89, *p* < 0.01) relative to the glycolysis/gluconeogenesis pathway. In the AcCKmi10 vs. AcTmi10 comparison group, miR-60-y (log_2_FC = 14.32, *p* < 0.01) potentially targeted an acs1p-encoding gene associated with the glycolysis/gluconeogenesis pathway (XM_002995904.1, log_2_FC = 3.78, *p* < 0.01) in the NcCKm vs. NcTm10 comparison group.

### Analysis of DEmRNAs in *Apis cerana cerana* workers’ midguts targeted by *Nosema ceranae* DEmiRNAs

**Figure 5:**
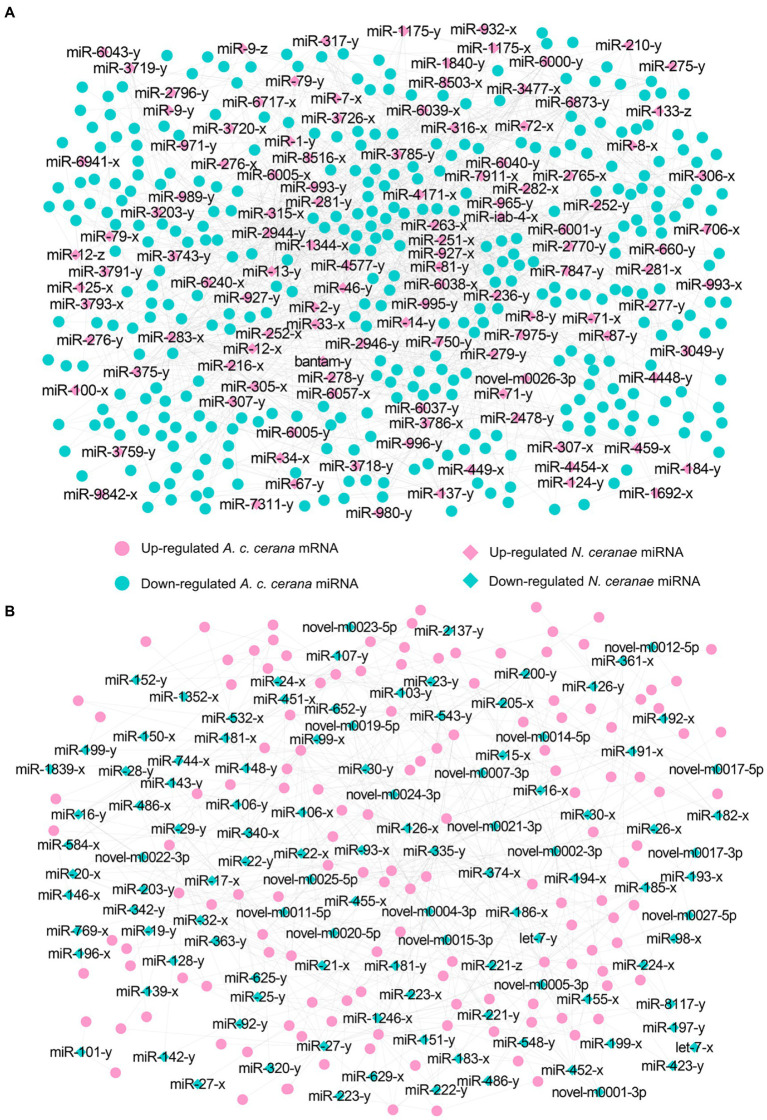
Regulatory networks of *N. ceranae* DEmiRNAs and target DEmRNAs in *A. c. cerana* workers’ midguts at 7 dpi. **(A)** Regulatory network between up-regulated miRNAs in microsporidian and down-regulated mRNAs in host. **(B)** Regulatory network down-regulated miRNAs in microsporidian and up-regulated mRNAs in host.

In total, 343 down-regulated mRNAs in AcCKm7 vs. AcTm7 were putative targets of 121 up-regulated miRNAs in NcCKmi vs. NcTmi7 (Figures 5A; see also Supplementary Table S2), while 138 up-regulated mRNAs of *A. c. cerana* in AcCKm7 vs. AcTm7 were potentially targeted by 112 down-regulated in NcCKmi vs. NcTmi7 (Figures 5B; see also Supplementary Table S2). The aforementioned 343 down-regulated mRNAs were related to 15 biological process-associated functional terms, including cellular process and metabolic process; 10 cellular component-associated functional terms, including cell and cell part; six molecular function-associated functional terms, including binding and catalytic activity (Supplementary Table S3); and 217 pathways, such as the Jak–STAT signaling pathway, endocytosis, and lysosome (Supplementary Table S4). Additionally, 138 up-regulated mRNAs were associated with 11 biological process-related functional terms, including cellular process, single-organism process, and metabolic process; eight cellular component-associated functional terms, including membrane part, membrane and organelle; seven molecular function-associated functional terms, including binding, catalytic activity and molecular transducer activity (Supplementary Table S3); and 107 pathways, including metabolic pathways, oxidative phosphorylation, and purine metabolism (Supplementary Table S4).

**Figure 6:**
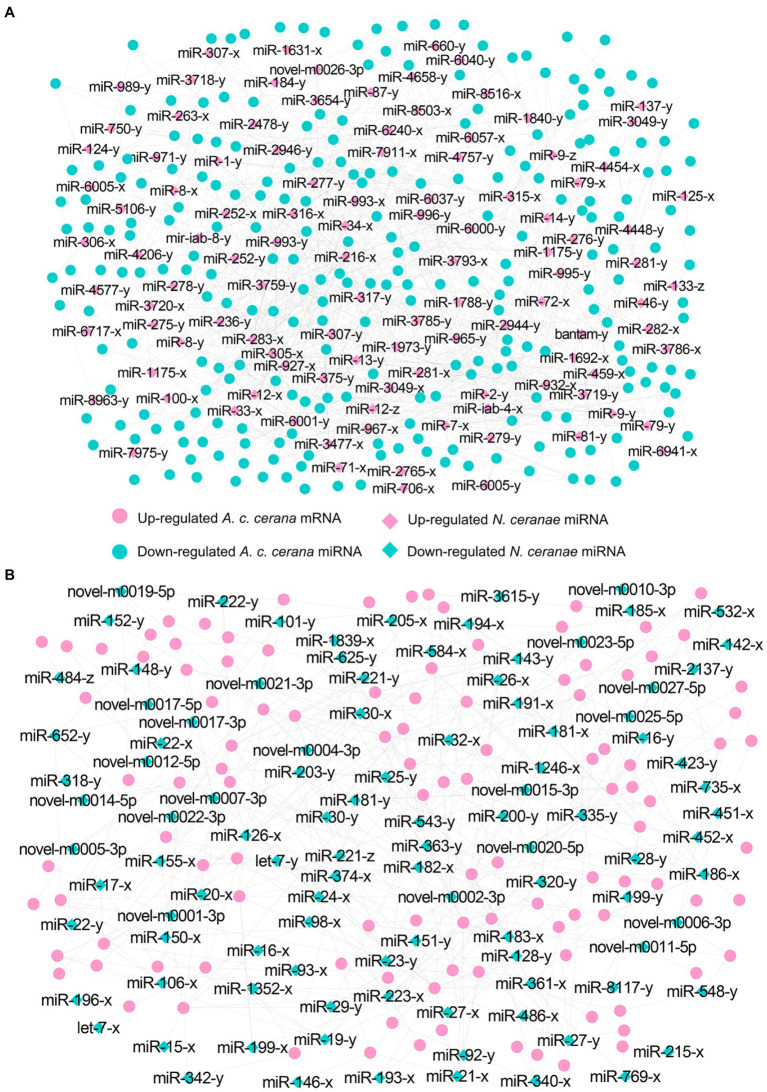
Regulatory networks of *N. ceranae* DEmiRNAs and target DEmRNAs in *A. c. cerana* workers’ midguts at 10 dpi. **(A)** Regulatory network between up-regulated miRNAs in microsporidian and down-regulated mRNAs in host. **(B)** Regulatory network down-regulated miRNAs in microsporidian and up-regulated mRNAs in host.

In the AcCKm10 vs. AcTm10 comparison group, 247 down-regulated and 110 up-regulated mRNAs were putatively targeted by 110 up-regulated and 104 down-regulated miRNAs in NcCKmi vs. NcTmi10 (Figures 6; see also Supplementary Table S2). The 104 down-regulated mRNAs were engaged in 23 functional terms, including cellular processes, membrane parts, and catalytic activity (Supplementary Table S3); and 142 pathways, including ubiquitin-mediated proteolysis, platinum drug resistance, and apoptosis-fly (Supplementary Table S4). Additionally, 110 up-regulated mRNAs were involved in 23 functional terms, including metabolic process, binding, and catalytic activity (Supplementary Table S3); and 89 pathways, including quorum sensing, folate biosynthesis, and platinum drug resistance (Supplementary Table S4).

**Figure 7:**
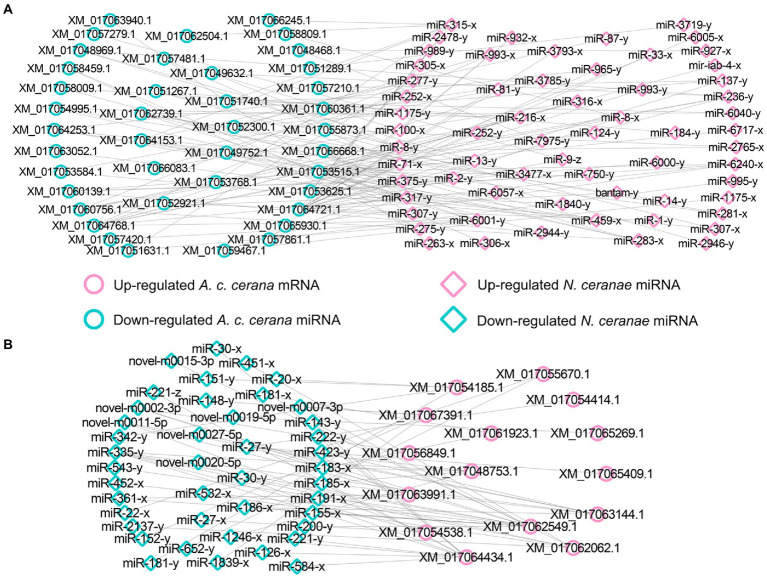
Regulatory networks of DEmiRNAs shared by NcCKmi vs. NcTmi7 and NcCKmi vs. NcTmi10 comparison groups and their targeted DEmRNAs shared by AcCKm7 vs. AcTm7 and AcCKm10 vs. AcTm10 comparison groups. **(A)** Network of common up-regulated miRNAs in microsporidian and common down-regulated mRNAs in host. **(B)** Network of shared down-regulated miRNAs in microsporidian and common up-regulated mRNAs in host.

Further investigation showed that 62 up- and 42 down-regulated miRNAs shared by the NcCKmi vs. NcTmi7 and NcCKmi vs. NcTmi10 comparison groups could potentially target 40 down-regulated and 15 common up-regulated mRNAs shared by the AcCKm7 vs. AcTm7 and AcCKm10 vs. AcTm10 comparison groups (Figure 7).

**Figure 8:**
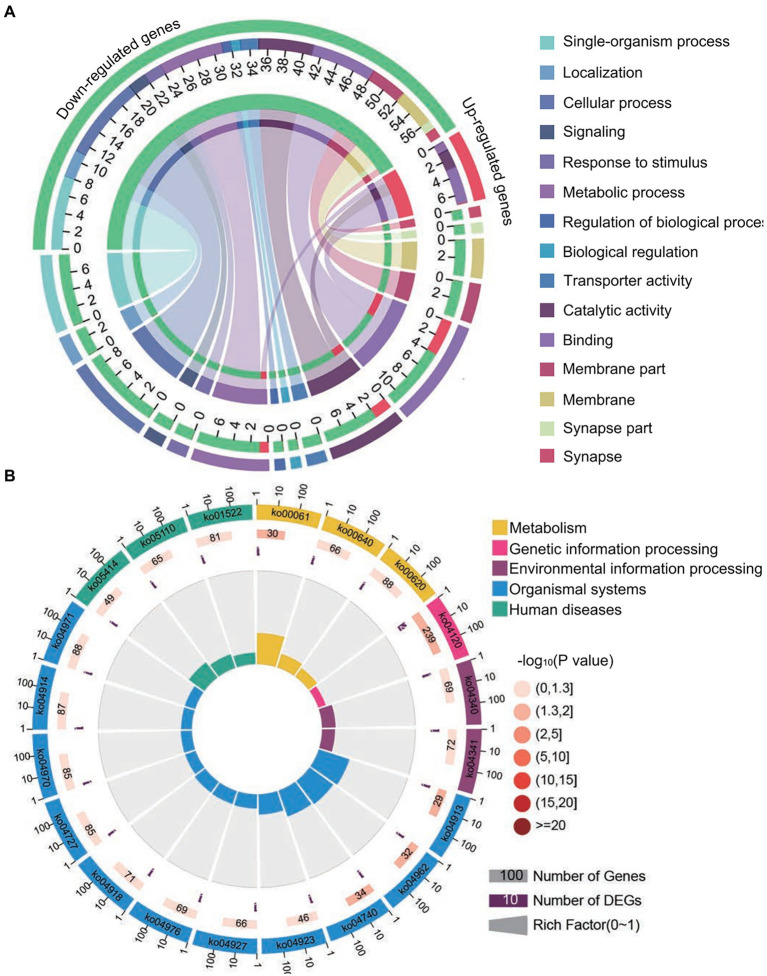
Database annotation of shared *A. c. cerana* DEmRNAs targeted by *N. ceranae* DEmiRNAs. **(A)** GO database annotation of DEmRNAs shared by AcCKm7 vs. AcTm7 and AcCKm10 vs. AcTm10. **(B)** KEGG database annotation of down-regulated mRNAs shared by AcCKm7 vs. AcTm7 and AcCKm10 vs. AcTm10.

Forty common down-regulated mRNAs in AcCKm7 vs. AcTm7 and AcCKm10 vs. AcTm10 targeted by 15 common up-regulated miRNAs in NcCKmi vs. NcTmi7 and NcCKmi vs. NcTmi10 were annotated to 18 GO terms; 12 mRNAs were engaged in biological process-associated categories, and the top three subcategories were cellular process, single-organism process, and metabolic process; four mRNAs were involved in cellular component-associated category, such as membrane part, membrane, cell, and cell part; and ten mRNAs were engaged in molecular function-associated categories, among which the largest group was binding followed by catalytic activity and transporter activity (Figure 8A; Supplementary Table S3). Additionally, the mRNAs mentioned above could be annotated to 70 pathways, such as fatty acid biosynthesis, ovarian steroidogenesis, and aflatoxin biosynthesis (Figure 8B; Supplementary Table S4).

### Investigation of *Nosema ceranae* DEmiRNAs and their target DEmRNAs associated with *Apis cerana cerana* immune- and energy metabolism-related pathways

**Figure 9:**
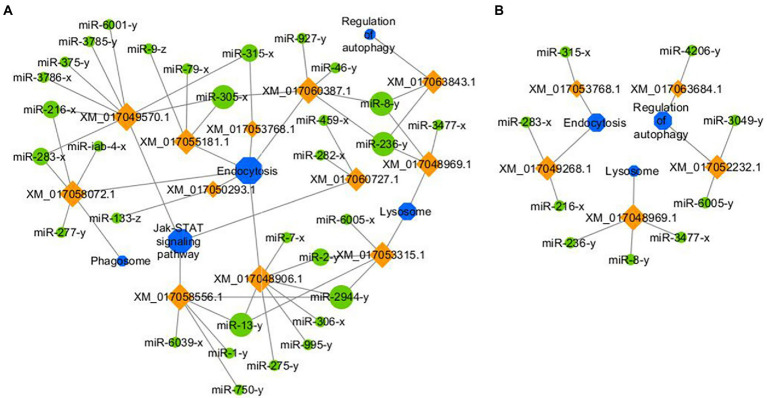
Regulatory networks of *N. ceranae* DEmiRNAs and their target DEmRNAs associated with *A. c. cerana* immune-related pathways. **(A)** Network of up-regulated miRNAs in NcCKmi vs. NcTmi7 and immune-associated down-regulated mRNAs in AcCKm7 vs. AcTm7. **(B)** Network of up-regulated miRNAs in NcCKmi vs. NcTmi10 and immune-associated down-regulated mRNAs in AcCKm10 vs. AcTm10.

Further investigation indicated that 31 up-regulated miRNAs in NcCKmi vs. NcTmi7 potentially targeted 12 down-regulated mRNAs in AcCKm7 vs. AcTm7 (Supplementary Table S5), which were involved in five immune-related pathways, endocytosis, lysosomes, phagosome, Jak–STAT signaling pathway, and regulation of autophagy (Figure 9A). Comparatively, nine up-regulated miRNAs in NcCKmi vs. NcTmi10 putatively targeted five down-regulated mRNAs in AcCKm10 vs. AcTm10 (Supplementary Table S5), which were engaged in three immune-related pathways, including endocytosis, lysosomes, and regulation of autophagy (Figure 9B). In addition, the down-regulated miR-21-x (log_2_FC = −12.51, *p* < 0.05) in NcCKmi vs. NcTmi7 potentially targeted an up-regulated mRNA encoding NADH dehydrogenase [ubiquinone] 1 alpha subcomplex subunit 5 (XM_017057571.1, log_2_FC = 1.28, *p* < 0.01) in AcCKm7 vs. AcTm7, which was relevant to oxidative phosphorylation, a key energy metabolism pathway (Supplementary Table S5).

### Validation of the targeting relationship between *Apis cerana cerana* miRNAs and *Nosema ceranae* mRNAs and between *Nosema ceranae* miRNAs and *Apis cerana cerana* mRNAs

**Figure 10:**
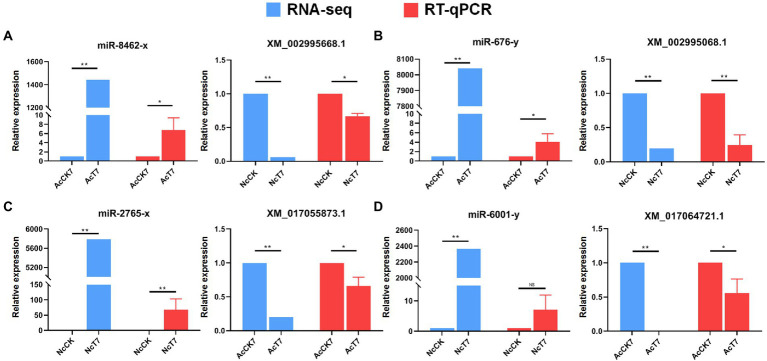
RT-qPCR validation of potential host miRNAs-microsporidian mRNAs and microsporidian miRNAs-host mRNAs targeting relationship. **(A)** RNA-seq and RT-qPCR results of *A. c. cerana* miR-8,462-x and *N. ceranae* XM_002995668.1. **(B)** RNA-seq and RT-qPCR results of *A. c. cerana* miR-676-y and *N. ceranae* XM_002995068.1. **(C)** RNA-seq and RT-qPCR results of *N. ceranae* miR-2,765-x and *A. c. cerana* XM_017055873.1. **(D)** RNA-seq and RT-qPCR results of *N. ceranae* miR-6,001-y and *A. c. cerana* XM_017064721.1.

Following the target prediction results in this study, two *A. c. cerana* DEmiRNAs (miR-8,462-x and miR-676-y) and corresponding target DEmRNAs in *N. ceranae* (XM_002995668.1 and XM_002995068.1) as well as two *N. ceranae* DEmiRNAs (miR-2,765-x and miR-6,001-y) and corresponding target DEmRNAs in *A. c. cerana* (XM_017055873.1 and XM_017064721.1) were randomly selected for RT–qPCR validation. The results indicated that the expression trends of the abovementioned four miRNAs and four mRNAs were consistent with those of the transcriptome data, and a negative relationship was observed between the *A. c. cerana* DEmiRNAs and *N. ceranae* DEmRNAs as well as *N. ceranae* DEmiRNAs and *A. c. cerana* DEmRNAs (Figure 10), which confirmed the reliability of our transcriptome data and the targeting relationship between the hosts and microsporidians.

**Figure 11:**
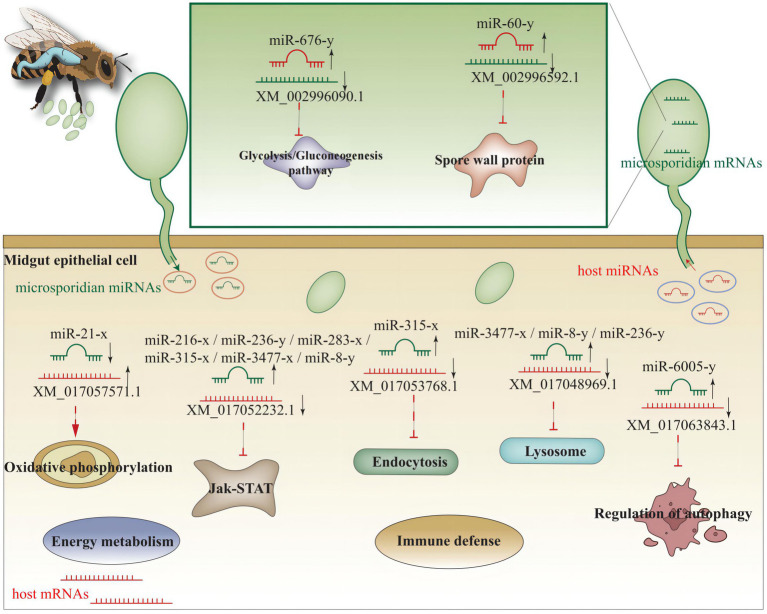
miRNA-mediated cross-kingdom regulation between *A. c. cerana* and *N. ceranae.*

Based on the findings in the present study, a working model of miRNA-mediated cross-kingdom regulation between *A. c. cerana* and *N. ceranae* was summarized in Figure 11.

## Discussion

### Potential negative regulatory relationships exist between *Apis cerana cerana* DEmiRNAs as well as *Nosema ceranae* DEmRNAs and vice versa

Currently, cross-kingdom regulation between insects and fungal pathogens is poorly understood. As key regulators in regulating gene expression and biological processes, miRNAs have been verified to mediate cross-kingdom regulation among animals, plants, and microorganisms (Halder et al., 2022; Rabuma et al., 2022). During the infection of *Solanum lycopersicum* and *Aedes albopictus*, *Botrytis cinerea* and *Beauveria bassiana* can synthesize and secrete sRNAs into the host cell *via* vesicles and hijack the RNA interference mechanism by binding to the Argonaute 1 protein, which further silenced the expression of host immune genes (Weiberg et al., 2013; Cui et al., 2019). In cotton infected by *Verticillium dahliae*, the host-derived miR159 and miR166 were transported to the fungal mycelium and specifically silenced the fungal virulence factors isotrichomycin C-15 hydroxylase and Ca^2+^-dependent cysteine protease encoding genes, rendering cotton somewhat resistance to *V. dahliae* (Zhang et al., 2016). Here, we conducted a comprehensive investigation of miRNA-mediated cross-kingdom regulation between *A. c. cerana* workers and *N. ceranae* for the first time by utilizing previously obtained high-quality transcriptome data. It is discovered that eight up-regulated miRNAs in *A. c. cerana* worker’s midgut at 7 dpi could target 97 down-regulated mRNAs in *N. ceranae* (Figure 1), while six down-regulated host miRNAs had 60 targets that up-regulated in microsporidian; seven up-regulated and two down-regulated miRNAs in host midgut at 10 dpi could target 44 down-regulated and 15 up-regulated *N. ceranae* mRNAs, respectively (Figure 2). This suggested that there were potential targeting relationships between *A. c. cerana* DEmiRNAs and *N. ceranae* DEmRNAs, more genes in *N. ceranae* were suppressed by host DEmiRNAs, implying a miRNA-mediated manner may be adopted by *A. c. cerana* workers to defense against microsporidian infection;however, some *N. ceranae* genes were induced to activation, indicating a putative strategy used by *N. ceranae* to manipulate expression of host miRNAs, which was reported in other pathogens and parasites such as *Schistosoma japonicum* (Wang et al., 2022) and *Botrytis cinerea* (Weiberg et al., 2013). In addition, 121 up-regulated miRNA in *N. ceranae* at 7 dpi could target 343 down-regulated host mRNAs, while 112 down-regulated *N. ceranae* miRNAs could target 138 up-regulated mRNAs in host midgut (Figure 5); comparatively, 110 up-regulated and 104 down-regulated *N. ceranae* miRNAs could, respectively, target 247 down-regulated and 110 up-regulated mRNAs (Figure 6), suggesting that miRNAs were probably utilized as a weapon to achieve the goal of host manipulation during *N. ceranae* infection. Intriguingly, considerable DEmiRNAs were employed by *N. ceranae* to regulate host gene expression though microsporidian was considered to be one of the most simplified eukaryotes (Nakjang et al., 2013), which implied the crucial role of miRNAs in microsporidia (Dong et al., 2021; Hu et al., 2021). Additional experimental work such as overexpression and knockdown are needed to verify function of *N. ceranae* miRNAs during the infection process. These results together were indicative of potential targeting relationships between *N. ceranae* DEmiRNAs and *A. c. cerana* DEmRNAs as well as between *A. c. cerana* DEmiRNAs and *N. ceranae* DEmRNAs.

It is generally accepted that miRNA directly mediates post-transcriptional gene silencing (PTGS) in the cytoplasm through binding of the “seed sequence” within a miRNA with the complementary sequences in the 3′-untranslated region (UTR) of target mRNA (Lewis et al., 2003; Bartel et al., 2009), which further induces mRNA cleavage or translational inhibition (Van den Berg et al., 2008). However, with the development of related studies, recent findings showed that some miRNAs could also trigger positive regulation of gene expression in a non-classical manner (Orom et al., 2008; Panda et al., 2014). For example, Shimakami et al. (2012) discovered that miR-122 can bind to the 5′ UTR of the positive-strand of Hepatitis C virus (HCV) RNA genome to slow down the decay of the viral genome in infected FT3-7 cells, further leading to the stimulation of viral protein expression and promotion of viral replication. In this current work, we also detected that 211 up-regulated *N. ceranae* miRNAs putatively targeted 238 up-regulated *A. c. cerana* mRNAs, which were relative to metabolism and cellular process; 16 up-regulated miRNAs of host target the 161 up-regulated mRNAs of microsporidian. Considering the major projective of this study was to investigate the classical regulatory manner of *N. ceranae* and *A. c. cerana* DEmiRNAs, only negative regulatory relationships between host DEmiRNAs and microsporidian DEmRNAs as well as between host DEmiRNAs and microsporidian DEmRNAs were analyzed and discussed. In the near future, we will further investigate the miRNA-mediated positive regulation and explore the underlying molecular mechanism.

### *Apis cerana cerana* DEmiRNAs putatively regulate glycolysis/glyconeogenesis and virulence factors in *Nosema ceranae*

With an extremely reduced genome, *N. ceranae* is unable to produce ATP *via* the tricarboxylic acid (TCA) cycle and oxidative phosphorylation, and is thus highly dependent on host cell-produced energy; however, *N. ceranae* retains the intact glycolysis pathway, which is active primarily in the spore stage (Dolgikh et al., 1997; Hoch et al., 2002; He et al., 2020). In this work, it is detected that host miR-676-y (log_2_FC = 12.97, *p* < 0.01) could target XM_002996090.1 (log_2_FC = −6.89, *p* < 0.01), which is associated with the glycolysis/gluconeogenesis pathway in *N. ceranae*, at 7 dpi (Figure 5); additionally, the host miR-60-y (log_2_FC = 14.32, *p* < 0.01) could target XM_002995904.1 (log_2_FC = 3.78, *p* < 0.01) in *N. ceranae* at 10 dpi (Figure 5). The results implied that miR-676-y and miR-60-y were potentially employed by the host to downregulate the expression of associated genes and impact the glycolysis/gluconeogenesis pathway, which may further inhibit the energy metabolism of *N. ceranae*.

Microsporidian cells are simplified and lack mitochondria but contain mitosome, a genome-less organelle, which appears to function in iron–sulfur biochemistry and oxidative phosphorylation (Burri et al., 2006). The thick spore wall of microsporidians is composed of proteins and chitins, which helps microsporidia survive in various harsh environments (Vávra, 1976). Spore wall proteins (SWPs) have also been verified to interact with host cells and participate in the microsporidia infection process (Jaroenlak et al., 2018; Yang et al., 2018). It is reported that NbSWP5 was involved in the germination of *N. bombycis* spores by interacting with polar tube proteins, and exerted function in protecting spores from phagocytic uptake by cultured insect cells (Cai et al., 2011; Li et al., 2012). The oral ingestion of *SWP12* dsRNAs caused a significant reduction of the *N. ceranae* spore load in *A. m. ligustica* workers’ midguts, while simultaneously improved host immune defense and lifespan (He et al., 2021). In *C. elegans*, miR-60 was exclusively expressed in the intestinal tissue, modulated the endocytosis machinery and zip-10-mediated innate immune systems, and coordinated the expression of genes engaged in cellular homeostasis, further promoting an adaptive response against long-term mild oxidative stress (Kato et al., 2016). Here, we found miR-60-y was significantly up-regulated (log_2_FC = 10.87, *p* < 0.01) in the midgut of *A. c. cerana* workers at 7 dpi, and could target the mRNA (XM_002996592.1, log_2_FC = −2.24, *p* < 0.01) encoding *SWP12* in *N. ceranae*, indicating that miR-60-y may be utilized by the host to suppress *N. ceranae* infection *via* negative regulation of the *SWP12* expression. Together, these results demonstrated that the aforementioned three host DEmiRNAs were potentially induced to activation to inhibit genes associated with glycolysis/glyconeogenesis and SWPs during *N. ceranae* infection.

Effective overexpression and knockdown of fungal miRNAs have been achieved using corresponding mimics. For example, Chakrabarti et al. (2020) found that chemically synthesized mimics of erythrocytic miR-150-3p and miR-197-5p were loaded into erythrocytes and subsequently used for invasion by the parasite, growth of *Plasmodium falciparum* was hindered in miRNA-loaded erythrocytes, and both micronemal secretion and *Apicortin* expression were reduced in miRNA-loaded erythrocytes. By treating RAW 264.7 cells infected by *Leishmania major* promastigotes (MRHO/IR/75/ER) with the miR-15a mimic and/or miR-155 inhibitor, Gholamrezaei et al. (2020) detected that apoptosis of macrophages was increased whereas the parasite burden was reduced. At present, cultured cells for *N. ceranae* are still lacking, thus limiting *in vitro* functional studies of *N. ceranae* genes. However, an *N. ceranae*-infected bee model has been constructed and applied to functional studies on microsporidian proliferation-associated genes. Huang et al. (2019) previously injected siRNA-*Dicer* into *A. mellifera* workers infected with *N. ceranae*, and observed that genes encoding two virulence factors, ABC transporter and hexokinase, were down-regulated while the spore load was significantly reduced, which indicated that *Dicer* gene silencing could suppress the *N. ceranae* proliferation. Recently, Kim et al. (2020) conducted dsRNA-based RNAi of mitosome-related genes in *N. ceranae*, and the results showed that microsporidian proliferation was inhibited while the host survival rate was increased. To clarify the function of the above-mentioned genes targeted by host miR-676-y and miR-60-y, siRNA- or dsRNA-based RNAi of these targets will be conducted in the near future. Cross-border RNAi has been successfully applied in crop breeding for disease resistance, so it may provide a new way to control microsporidiosis by interfering with the absorption of miR-676-y, etc. by *N. ceranae*.

### *Nosema Ceranae* DEmiRNAs potentially regulate the energy mechanism and immune response of *Apis cerana cerana* workers

Microsporidia are a group of obligate intracellular parasites that lack mitochondria; hence, microsporidia evolve unique strategies to acquire nutrients from host cells and manipulate host metabolism (Yang et al., 2018). By analyzing the miRNA response of *A. mellifera* workers to *N. ceranae* infection, Evans and Huang (2018) uncovered that *N. ceranae* DEmiRNAs may regulate the expression of host genes relevant to purine metabolism, pyrimidine metabolism, and oxidative phosphorylation. In the present study, miR-21-x (log_2_FC = −12.51, *p* < 0.05) was significantly down-regulated at 7 dpi and potentially targeted the XM_017057571.1(log_2_FC = 1.13, *p* < 0.01), an up-regulated mRNA involved in host oxidative phosphorylation (Figure 9; Supplementary Table S5), indicating the activation of the host energy mechanism by microsporidians. These findings suggested that oxidative phosphorylation in host cells was activated by *N. ceranae* to provide more ATP for microsporidian proliferation.

Similar to other insects, honey bees have evolved an innate immune system to fight against foreign invaders (Evans et al., 2006). Insect innate immunity consists of three main processes: (1) pathogen recognition; (2) signal transduction; and (3) immune signaling pathway, downstream gene expression, and final immune response regulation (Naitza and Ligoxygakis, 2004). The insect immune system can be divided into cellular and humoral immunity (McMenamin et al., 2018); however, these two kinds of immunity cannot be strictly separated since both are interrelated and act together to generate highly effective defenses (Li et al., 2018). Humoral immunity senses a wide range of pathogens through distinct pattern recognition receptors (PRRs), and the engagement of these PRRs induces the activation of specific signaling pathways, leading to the expression of antimicrobial genes (McMenamin et al., 2018). The JAK/STAT signaling pathway plays a central role in the immune response of bee individuals (McMenamin et al., 2018). In silkworms, a weak response of the JAK/STAT signaling pathway could be induced by fungal infection (Chen et al., 2018). Antúnez et al. (2009) detected that the immune response of *A. m. ligustica* workers was suppressed at 7 dpi with *N. ceranae*, and the expression of host genes encoding antimicrobial peptides and immune-related enzymes was significantly down-regulated. Here, a total of 15 up-regulated *N. ceranae* miRNAs were found to target three down-regulated mRNAs enriched in the Jak/STAT signaling pathway in host midgut at 7 dpi. Additionally, miR-216-x (NcCKmi vs. NcTmi7: log_2_FC = 14.92, *p* < 0.01; NcCKmi vs. NcTmi10: log_2_FC = 15.10, *p* < 0.01), miR-236-y (NcCKmi vs. NcTmi7: log_2_FC = 12.98, *p* < 0.01; NcCK vs. NcTmi10: log_2_FC = 12.96, *p* < 0.01), miR-283-x (NcCKmi vs. NcTmi7: log_2_FC = 9.41, *p* < 0.05; NcCK vs. NcTmi10: log_2_FC = 9.08, *p* < 0.05), miR-315-x (NcCKmi vs. NcTmi7: log_2_FC = 17.11, *p* < 0.01; NcCKmi vs. NcTmi10: log_2_FC = 16.33, *p* < 0.01), miR-3,477-x (NcCKmi vs. NcTmi7: log_2_FC = 22.22, *p* < 0.01; NcCKmi vs. NcTmi10: log_2_FC = 21.73, *p* < 0.01), and miR-8-y (NcCKmi vs. NcTmi7: log_2_FC = 9.68, *p* < 0.05; NcCKmi vs. NcTmi10: log_2_FC = 9.54, *p* < 0.05) were up-regulated in the aforementioned two comparison groups, and potentially targeted corresponding down-regulated mRNA relevant to immune pathways, such as XM_017052232.1 (log_2_FC = −11.13, *p* < 0.01) and XM_017049268.1 (log_2_FC = −12.02, *p* < 0.01; Figure 9; Supplementary Table S5).

The *A. mellifera* genome contains genes encoding most members of insect immune pathways, including orthologs of genes engaged in autophagy and endocytosis (McMenamin et al., 2018). Endocytosis is a dynamic process that both positively and negatively regulates various signaling pathways (Papagiannouli, 2022). Lysosomes have antifungal and antiviral activities and dissolve pathogenic proteins delivered by endocytosis and phagocytosis (Samaranayake et al., 2001). Here, we observed that miR-315-x (NcCKmi vs. NcTmi7: log_2_FC = 17.11, *p* < 0.01; NcCKmi vs. NcTmi10: log_2_FC = 16.33, *p* < 0.01) was down-regulated in *N. ceranae* at both 7 dpi and 10 dpi, and targeted the XM_017053768.1 (NcCKmi vs. NcTmi7: log_2_FC = −9.97, *p* < 0.01; NcCKmi vs. NcTmi10: log_2_FC = −9.44, *p* < 0.01) which was relevant to endocytosis. In additionally, miR-3,477-x (NcCKmi vs. NcTmi7: log_2_FC = 22.22, *p* < 0.01; NcCK vs. NcTmi10: log_2_FC = 21.73, *p* < 0.01), miR-8-y (NcCKmi vs. NcTmi7: log_2_FC = 9.68, *p* < 0.05; NcCKmi vs. NcTmi10: log_2_FC = 9.54, *p* < 0.05), and miR-236-y (NcCKmi vs. NcTmi7: log_2_FC = 12.98, *p* < 0.01; NcCKmi vs. NcTmi10: log_2_FC = 12.96, *p* < 0.01) were up-regulated in both NcCKmi vs. NcTmi7 and NcCKmi vs. NcTmi10 comparison groups, and putatively targeted the same mRNA XM_017048969.1 (NcCKmi vs. NcTmi7: log_2_FC = −11.47, *p* < 0.01; NcCKmi vs. NcTmi10: log_2_FC = −10.21, *p* < 0.05), a lysosome-associated gene. These findings suggested that *N. ceranae* may up-regulate the expression of the abovementioned host-derived miRNAs to weaken humoral and cellular immunity during infection.

## Conclusion

Taken together, we deciphered the landscape of DEmRNA-mediated cross-kingdom regulation between *A. c. cerana* workers and *N. ceranae* for the first time, and revealed that host-generated DEmiRNAs probably regulate glycolysis/glyconeogenesis and virulence factors during *N. ceranae* infection to suppress microsporidian proliferation while *N. ceranae*-derived DEmiRNAs were likely to enhance the host energy mechanism and at the meantime inhibit host immune responses to facilitate microsporidian invasion. Findings in this current work lay a foundation for exploring the mechanism underlying cross-kingdom regulation between *A. c. cerana* workers and *N. ceranae*, provide valuable insights into Asian honey bee-microsporidian interactions, and offer potential targets for bee nosemosis control.

## Data availability statement

The datasets presented in this study can be found in online repositories. The names of the repository/repositories and accession number(s) can be found in the article/Supplementary material.

## Author contributions

RG and DC: conceptualization. XF, WZ, KYZ, JZ, QL, KHZ, and LZ: methodology and data and investigation. XF, WZ, DC, and RG: original draft preparation. DC and RG: review and editing of the manuscript. RG, DC, XF, WZ, KYZ, JZ, QL, YW, KHZ, and LZ: supervision. All authors contributed to the article and approved the submitted version.

## Funding

This work was financially supported by the National Natural Science Foundation of China (32172792), the Earmarked fund for CARS-44-KXJ7 (CARS-44-KXJ7), the Master Supervisor Team Fund of Fujian Agriculture and Forestry University (RG), the Scientifc Research Project of College of Animal Sciences (College of Bee Science) of Fujian Agriculture and Forestry University (RG), the National Undergraduate Innovation and Entrepreneurship Training Program (KHZ), and the Undergraduate Innovation and Entrepreneurship Training Program of Fujian province (202210389128).

## Conflict of interest

The authors declare that the research was conducted in the absence of any commercial or financial relationships that could be construed as a potential conflict of interest.

## Publisher’s note

All claims expressed in this article are solely those of the authors and do not necessarily represent those of their affiliated organizations, or those of the publisher, the editors and the reviewers. Any product that may be evaluated in this article, or claim that may be made by its manufacturer, is not guaranteed or endorsed by the publisher.

## Footnotes

^1^ http://www.ncbi.nlm.nih.gov/sra/

^2^ http://www.omicshare.com/tools/
